# Is sexual attraction and place of origin a moderator of sex in pornography consumption? Cross-sectional study on a representative sample of young adults

**DOI:** 10.1186/s12889-023-16216-3

**Published:** 2023-07-13

**Authors:** Belén Sanz-Barbero, Vanesa Pérez-Martínez, J. Francisco Estévez-García, Carmen Vives-Cases

**Affiliations:** 1 National School of Public Health, Instituto de Salud Carlos III, Madrid, Spain; 2 CIBER of Epidemiology and Public Health (CIBERESP), Madrid, Spain; 3 Community Nursing, Preventive Medicine and Public health and History of Science Department, University of Alicante, Alicante, Spain; 4 Sociology II Department, University of Alicante, Alicante, Spain

**Keywords:** Pornography, Young people, Sexual attraction, Country of birth

## Abstract

**Background:**

Pornography consumption is higher in men, but we do not know if this association can be modified by different variables, such as sexual attraction and place of origin. Given the impact pornography has on minors, there are limited studies that analyze the use of pornography in representative samples of the adult population. The aim was analyze the prevalence and factors associated with using pornography in young adult men and women, living in Spain, with different sexual attractions and different places of birth.

**Methods:**

Cross-sectional study with an online survey conducted with 2515 men and women aged between 18 and 35 years of age. The prevalence of pornography consumption is described and analyzed in the total sample and stratified by sex, according to socio-demographic and sexual attraction variables. The association between covariates and pornography consumption at some point in life was estimated with prevalence ratios (PR) obtained with the Poisson models of robust variance. Dependent variable: voluntarily using pornography at some point in life. Socio-demographic variables were included in the analysis: age, sex, level of education, place of birth. Sexual attraction was also analyzed.

**Results:**

In Spain, 94.7% of men between 18 and 34 years and 74.6% of women have voluntarily used pornography at some point in their life. The mean age to start using it is earlier in men [Mean:14.2; Standard Deviation (SD):2.3]. Bisexual/homosexual attraction (reference: heterosexual) increases the probability of using pornography in women [(PR (95%CI): 1.30 (1.22; 1.38)]. Yet this is not observed in men. In both sexes, the probability of using pornography increases with age [(PR (95%CI): 1.01(1.00; 1.01)] and coming from abroad (reference: native), being the effect of country of birth significantly higher in women [(PR (95%CI): 1.17 (1.09; 1.26)] than in men [(PR (95%CI): 1.04 (1.01; 1.07).

**Conclusions:**

Public health programmes aimed at improving affective-sexual health should consider the high use of pornography among young adults in Spain, as well as those variables that increase its use.

## Background

The development of digital technologies over the last decades has prompted an almost unlimited amount of sexually explicit material to which people can access anonymously and for free [1]. In 2021, PornHub, one of the most popular pornography websites in the world, recorded 130 million daily visits. Moreover, 26% of said visits were from Spain, which is the geographic scope of this study [2].

The increased presence and accessibility of pornography in the digital sphere in recent decades has resulted in a large body of literature analyzing both the content of pornography [3, 4], user profile [5, 6], reasons for use [7] and the association of using pornography with affective-sexual thoughts, attitudes and behaviours [8–10]. A greater acceptance of violence and rape myths [10], more positive attitudes towards sexual coercion [11] as well as greater acceptance of violence against women by both sexes [10, 12] has been identified in pornography users. Despite these results, there are also studies showing that users of pornography sometimes consider it a non-harmful way of exploring pleasure and sexuality [13].

Although acts of explicit violence are not systematically present in pornography, symbolic violence is recurrent [14, 15]. By analyzing the most viewed online pornographic content, pornography focused on male pleasure, which represents women as a sexual object, depersonalized, fragmented and at the service of men’s desires is observed [16]. Humiliation, abuse and coercion towards women are frequent in pornography, as well as aggressive behaviours that are eroticized and to which women succumb and respond with displays of pleasure in pornography [8, 16].

Given the high impact that watching pornography can have on the affective-sexual development of younger people [17], many studies have focused on children and adolescents [17], with less analyzing adults [13, 18, 19]. Some of the reasons that lead to young people using pornography are curiosity, searching for sexual arousal and searching for information on sexual practices [20].

The prevalence of pornography use shows variability, with few studies reporting prevalence based on representative samples [18, 21, 22]. The majority of studies place prevalence of use around 85–95% in men and around 40–70% in women, both in adolescents [22] and young adults [23, 24]. In Spain, recent studies show that 98% of men and 88% of women between 18 and 25 years of age have used online pornography at some point in their life. This consumption does not significantly decrease with age both in men and women [7].

The association of sex with the use of pornography has been widely evidenced with a higher prevalence, greater frequency and earlier use among men than among women [6, 7, 18, 25, 26]. However, information on whether this association can be modified according to sociodemographic variables like sexual attraction or immigration status is limited, inconclusive and heterogeneous. Thus, most of the studies identify a greater use of pornography in homosexuals/bisexuals than in heterosexuals [16, 17, 21], but there is no consolidated evidence on the possible moderating role of sex regarding the association of sexual attraction with the use of pornography. Some studies identify an independent effect of these variables [25], while there are others that identify an association between sexual attraction and the use of pornography in men, but not women [20, 27]. Other variables, such as migratory status or country of birth and their association with using pornography, have been scarcely analyzed in comparison to ethnicity where different studies confirm the relation with consuming pornography. This is more frequent and probable in ethnic minorities [28, 29].

Identifying pornography user profiles has important consequences for public health. Both cross-sectional and longitudinal studies recognize an association between pornography use and victimization [11, 30] and perpetration of sexual violence [31, 32]. This association is greater when exposed to violent pornography [12, 32, 33], although there are studies that have not identified this association or display contradictory results [34, 35]. The utilization of pornography among adolescents has been linked to an increased prevalence of risky sexual behavior [36]. Furthermore, in young adults exhibiting problematic pornography consumption, a correlation has been observed with compromised sexual health, characterized by an increased probability of experiencing erectile dysfunction and reduced sexual satisfaction [37, 38]. In men, a relationship has been identified between dissatisfaction with sexual body image and the use of Internet pornography [39]. On the other hand, these results, there are also studies showing that users of pornography sometimes consider it a non-harmful way of exploring pleasure and sexuality [13]. In Spain, studies on the use of pornography are limited. The majority of them describe the pornography user, the reasons for watching pornography [7] or they analyze the problems of pornography consumption [40]. Identifying the variables associated with using pornography in the younger population (considering sex, sexual attraction, place of origin and socio-economic level) can be useful to improve the content and effectiveness of public health programmes on affective-sexual health. The aim of this study is to analyze the prevalence and factors associated with the use of pornography in young adult men and women of different sexual attractions and country of origin, but residents in Spain. The study is based on the hypothesis that the use of pornography among young adults is high and is defined both by sex, with a greater use of men, and by sexual attraction, with a greater use in homosexuals/bisexuals. Other sociodemographic characteristics of interest not explored in depth in other studies are included, such as country of birth.

## Methods

Cross-sectional study using an online survey with men and women aged 18–35 residing in Spain. Detailed information on the methodology has been previously published [41]. All the participants had literacy skills. The database includes a total of 2515 records. The sample was collected from a closed panel that include 138,393 adults over the age of 16. The aim of the study design was to be representative of the non-institutionalized youth population between the ages of 18 and 35 residing in Spain, according to age, sex. Voluntary recording was not allowed. Panellists were invited by email to complete the survey. Those who accepted received (via email) an individual link to complete the online survey. Panellists received only non-survey specific incentives through a point-based rewards programme, which they could use to purchase products from different stores. Once an initial pilot study was carried out (30th September − 1st October 2020), information collection was then conducted between 15th and 28th October 2020. The response rate was 62.3%. Once the database was refined, it included information from 2,346 people (93.9%).

### Variables

#### Main outcome

The analyzed dependent variable considers whether pornography has been consumed at some point in life. This variable was collected by asking: *have you voluntarily watched pornography at some point in life?* Response categories were yes/no.

Furthermore, the age at which the people began to voluntarily watch pornography was collected by asking: *when did you voluntarily watch pornography for the first time?* (Continuous variable).

#### Covariates

Based on previous articles [13, 17, 25], sociodemographic variables and sexual attraction were included as covariates.

Socio-demographic variables included: sex (man, woman), age (continuous variable), highest completed level of studies (literacy skills but no education finished, primary, secondary, further education), country of birth (Spain, abroad). People who claimed to have been born abroad were asked for the country of birth. To mitigate language-related biases, our panel specifically comprised Spanish-speaking Latin American immigrants. As all the immigrant people were born in a Latin-American country, so the variable was codified in Spain/Latin-America. Sexual attraction was collected by asking: *With which of the following statements do you feel most identified? I am only attracted to women/I am usually attracted to women, but sometimes I am also attracted to men/I am equally attracted to women and men/I am usually attracted to men, but sometimes I am also attracted to women/I am only attracted to men/I am not attracted to women or men/No answer.* People who stated that they were exclusively attracted to people of the same sex were included in the homosexual attraction category (gay/lesbian, according to sex); people who only claimed to be attracted to people of the other sex were classified as heterosexual. People attracted to both sexes were included in the bisexual attraction category. People who responded that they were not attracted to either sex (n = 6) were omitted from the analysis due to the small size of this group. For the regression analysis, which we describe in the next section, the variable was categorized as heterosexual/homo-bisexual.

### Data analysis

First of all, the total sample is described, stratified by sex according to the previously described covariates. Below is a description of the prevalence of pornography consumption at some point in life in the total sample, stratified by sex according to socio-demographic, sexual attraction and age they started watching pornography variables. The differences in pornography consumption were obtained with the Chi-squared test. The mean differences in the age of starting to consume pornography between men and women were obtained using the F statistic derived from the analysis of variance (ANOVA).

The association between covariates and pornography use at some point in life was estimated using robust variance Poisson models. Crude and subsequently adjusted prevalence ratios were calculated. In order to obtain the adjusted model, the variables that were significant in the raw model were included one by one. Regardless of the statistical significance, the models were adjusted according to level of education given their association with the use of pornography identified by other authors [13].

Interactions between sex and covariates included in the models were explored. Given the presence of interactions, the global sample model with the interactions is presented, as well as the models corresponding to the sample stratified by sex.

## Results

The analyzed sample includes a total of 2346 records. Table 1 describes the total sample and stratified by sex, according to the socio-demographic and sexual attraction variables. Mean age of the surveyed people was 27.5 years. 50.2% were women, of which 11.9% had been born abroad, with 99% of the people being from Latin America. 23.9% of the surveyed women referred to being bisexual and 2.1% were only attracted to women. Regarding men, 12.7% indicate being attracted to both sexes and 10.2% are attracted only to men.

**Table 1:** Description of total sample and stratified by sex. Socio-demographic, sexual attraction and pornography consumption characteristics

	Total			Women			Men	
	n	%		n	%		n	%
**Highest completed level of studies**								
No studies completed-secondary studies	714	30.4		321	27.3		393	33.6
Superior	1632	69.6		856	72.7		776	66.4
**Country of birth**								
Abroad (latin)	280	11.9		165	14.0		115	9.8
Spain	2066	88.1		1012	86.0		1054	90.2
**Sexual attraction**								
Homosexual	144	11.3		25	2.1		119	10.2
Bisexual	430	18.3		281	23.9		149	12.7
Heterosexual	1772	75.5		871	74.0		901	77.1
	**n**	**Mean (SD)**		**n**	**Mean (SD)**		**n**	**Mean (SD)**
**Mean age (SD)**	2346	27.5 (4.6)		1177	27.5 (4.5)		1169	27.6 (4.8)
**Total**	2346	1000.0		1177	50.2		1169	49.8

N: frecuency; SD: standard deviation

Table 2 describes pornography consumption at some point in life, in the total sample and stratified by sex. The frequency of pornography consumption was significantly higher in men (94.7% men; 74.6% women), in people born abroad, both women (foreign women: 85.5% vs. 72.8% Spanish women) and men (foreign men: 98.3% vs. 94.3% Spanish men), as well as non-heterosexual women (homosexual/bisexual: 89.9% vs. heterosexual: 69.2%). The mean age of when they started to watch pornography was 15.4 years of age (SD 3.5), with this age being lower in men (14.2 years) (SD 2.3) than women (17.3 years; SD 4.2).

**Table 2:** Description of pornography consumption at some point in life. Total sample and stratified by sex

	Total sample				Women				Men		
	n	% consumption	*p*		n	% consumption	*p*		n	% consumption	*p*
**Highest completed level of studies**			0.45				0.148				0.061
No studies completed-secondary studies	598	83.8			232	72.3			366	93.1	
Superior	1387	85.0			646	75.5			741	95.5	
**Country of birth**			p < 0.01				p < 0.001				p < 0.05
Abroad (latin)	1731	83.8			141	85.5			113	98.3	
Spain	254	90.7			737	72.8			994	94.3	
**Sexual attraction**			p < 0.001				p < 0.001				0.122
Homosexual/bisexual	533	92.9			275	89.9			258	96.3	
Heterosexual	1452	81.9			603	69.2			849	94.2	
	**n**	**Mean (SD)**			**n**	**Mean (SD)**			**n**	**Mean (SD)**	
**Mean age (SD)**	1985	27.6 (4.6)	p < 0.01		878	27.6 (4.4)	0.06		1107	27.21 (4.7)	p < 0.05
**Mean age of initation of pornography consumption ever in a lifetime (SD) (na = 735)**	1250	15.43 (3.5)				17.3 (4.2)				14.2 (2.3)	p < 0.001
**Total**	1985	84.6			878	74.6			1107	94.7	p < 0.001

N: frecuency; SD: standard deviation; na: not answer

Tables 3 and 4 show the variables associated with pornography consumption in the total sample and stratified by sex. Table 3 includes the crude prevalence ratios and Table 4 the adjusted prevalence ratios.

**Table 3:** Variables associated with the use of pornography at some point in life. Poisson regression model with robust variance. Crude prevalence ratios

	Total sample					Women					Men			
	PRu	(CI 95%)		*p*		PRu	(CI 95%)		*p*		PRu	(CI 95%)		*p*
**Sex (Unexposed group: Women)**														
Men	1.27	(1.22	1.32)	p < 0.001										
**Age**	1.01	(1.00	1.01)	p < 0.010		1.01	(1.00	1.01)	0.06		1.01	(1.00	1.01)	p < 0.05
**Highest completed level of studies (Unexposed group: No studies completed-secondary studies)**														
Superior	1.01	(0.98	1.05)	0.453		1.04	(0.97	1.13)	0.276		1.03	(0.99	1.06)	0.113
**Country of birth (Unexposed group: Spain)**														
Abroad (latin america)	1.08	(1.04	1.13)	p < 0.001		1.17	(1.09	1.26)	p < 0.001		1.04	(1.01	1.07)	p < 0.01
**Sexual attraction (Unexposed group: Heterosexual)**														
Homosexual/bisexual	1.13	(1.10	1.17)	p < 0.001		1.30	(1.22	1.38)	p < 0.001		1.02	(0.99	1.05)	0.142

PRu: undajusted prevalence ratio; CI: confidence interval

**Table 4:** Variables associated with the use of pornography at some point in life. Poisson regression model with robust variance. Adjusted prevalence ratios

	Total sample					Women					Men			
	PRa	(CI 95%)		*p*		PRa	(CI 95%)		*p*		PRa	(CI 95%)		*p*
**Sex (Unexposed group: Women)**														
Men	1.01	(1.00	1.01)	p < 0.010		-	-	-	-		-	-	-	-
**Age**	1.01	(1.00	1.01)	p < 0.010		1.01	(1.00	1.02)	p < 0.05		1.00	(1.00	1.01)	p < 0.050
**Highest completed level of studies (Unexposed group: No studies completed-secondary studies)**														
Superior	1.03	(1.00	1.07)	0.077		1.06	(0.98	1.15)	0.138		1.02	(0.99	1.05)	0.275
**Country of birth (Unexposed group: Spain)**														
Abroad (latin america)	1.17	(1.09	1.26)	p < 0.001		1.17	(1.09	1.26)	p < 0.001		1.04	(1.01	1.07)	p < 0.050
**Sexual attraction (Unexposed group: Heterosexual)**														
Homosexual/bisexual	1.31	(1.23	1.38)	p < 0.001		1.31	(1.24	1.39)	p < 0.001		1.02	(0.99	1.05)	0.120
Constant	0.66	(0.62	0.70)	p < 0.001		0.65	(0.60	0.70)	p < 0.001		0.93	(0.90	0.95)	p < 0.001
***Interactions***														
**Country of birth x Sex**														
Foreign man	0.88	(0.82	0.96)	p < 0.010										
**Sexual attraction x Sex**														
Homosexual/bisexual man	0.78	(0.73	0.84)	p < 0.001										

PRa: adjusted prevalence ratio; CI: confidence interval

**Fig. 1:**
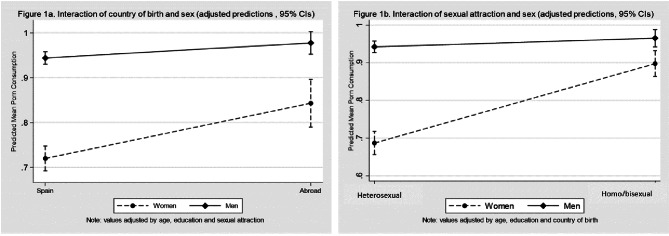
Prediction of pornography use according to the interaction of the sex variable with either country of birth or sexual attraction

As observed in Table 4, as age increases, the probability of pornography consumption rises, both in women [PR:1.01 IC 95% (1.00;1.02)] and men [PR:1.01 IC 95% (1.00;1.01)]. Two interactions were identified in the model, one between sex and country of birth (p < 0.01) and another between sex and sexual attraction (p < 0.001). In particular, an observation is made in the interaction of sex with country of origin (Table 4; Fig. 1a) that the probability of pornography consumption is significantly higher in people born abroad than in people born in Spain. This difference is significantly greater in women [PR: 1.17 IC95% 1.09;1.26)] than men [PR: 1.04 IC95% 1.01;1.07)]. A second interaction between sex and sexual attraction (Table 4; Fig. 1b) indicates that the probability of pornography consumption is greater in homosexual/bisexual women than heterosexual women [PR: 1.31 IC95% 1.24;1.39)], although this association between sexual attraction and pornography consumption is not seen among men [PR: 1.02 IC95% 0.99;1.05)].

## Discussion

In Spain, nine out of ten men between 18 and 34 years of age and seven out of ten women in the same age range have voluntarily used pornography at some point in their life. The mean age of when they started consuming pornography is earlier in men than women, around 14 and 17 years of age, respectively. In women, bisexual and homosexual sexual attraction increases the probability of pornography use regarding heterosexual women. This association has not been identified in men. Regarding both men and women, the probability of using pornography increases with age and with the fact of being born abroad, with the effect of place of birth being significantly greater in women than in men.

The prevalence of pornography consumption at some point in life described in our study is similar to that recently identified by Ballester [7] in young Spanish adults (18–25 years old), where 98% of men and 88% of women report to having consumed online pornography. In an international context, the data is more variable, varying between 14% and 82% in women and between 63% and 100% in men [18, 25]. The different time frame of the studies, the cultural differences in the population, as well as the methodological differences between them could partly explain this heterogeneity. Despite this, the results, as in our work, are consistent in identifying a greater or earlier exposure to pornography in men and women [6, 7, 18, 25, 26]. The fact that pornography is primarily aimed at seeking pleasure in men may explain this different frequency and motivation for pornography use. In turn, this greater use of pornography among men could be a reflection of how a different socialization of men and women on gender roles is projected into a different sexuality development. Regarding men, socialization in gender roles promotes the searching for pleasure and sexual experiences, power and sexual control that coincide both with the aims of pornography as well with the use men make of. In women, socialization based on gender roles promotes to a greater extent commitment in affective-sexual relationships, empathy, interpersonal care, values that are far from those found in pornography contents [42]. In this sense, the reasons leading to using digital media for sexual purposes are different for men and women [6, 7]. Men refer to a greater extent than women seeking sexual arousal, overcoming stressful situations and/or boredom, carrying out and observing behaviours that cannot be done in their real life, as well as meeting people with whom they can have sexual contact outside the virtual environment. Women refer to a greater extent than men using pornography to learn about sex and sexuality [6, 7].

The age when people start using pornography identified in our study is similar to that identified by other authors [7, 25, 26]. An earlier use is confirmed by men, compared to women, despite men developing later. This is possibly a reflection of how the different levels of socialization between men and women influences affective-sexual development, as previously explained.

Regarding sexual attraction, evidence on the association between sexual attraction and using pornography is heterogeneous. The majority of studies confirm a greater use among homosexuals and bisexuals [18, 21, 43, 44], although there are also other studies that do not observe this association [26]. Our results identify an association between sexual attraction and the use of pornography that was statistically significant in women, but not in men. The recently published study by Giménez García [43] with young adult Spanish women shows how homosexual and bisexual women use pornography more than heterosexual women. In an international context, studies by Traen [18] evidence a positive association between homosexual and bisexual attraction and pornography consumption. However, we do not know if this association is moderated or not by sex, as observed in our results. Contrary to our results, the work published by Luder [27] conducted with Swiss teenagers, identified a higher probability of pornography use in homosexual and bisexual boys than in heterosexuals. This association was not identified in girls. There is a chance that some of these differences in the results can be related to the temporal and geographical framework of the studies, as well as changes in sexuality experiences in this period. In this sense, in our study, 24% of women indicate to having a sexual attraction to both sexes, while the latest National Sexual Health Survey carried out in Spain in 2009, this percentage among women between 35 and 44 years of age was 4.3% [45]. This greater recognition at present in bisexual women, as well as the greater probability of using pornography in non-heterosexual attractions, could reflect an unmet need for affective-sexual education that incorporates sexual attraction in its contents.

A new aspect of our work is studying the association of place of birth with consuming pornography. Our results show that the probability of using pornography is significantly higher in people born abroad than those born in Spain. This difference is also substantially higher in women than men. Studies by Mahapatra show the increased use of pornography amongst immigrant men [28], but to the best of our knowledge, there are no studies that analyze the association of immigration status on the use of pornography, as well as its possibility interaction with sex. The fact that using pornography increases among people who have been away from their place of origin for a long time [28], and the greater usage probability among ethnic minorities in the USA [29], could indicate that there is a higher predisposition amongst this population possibly associated with situations of isolation, discrimination and stress. In this sense, facing stress, loneliness and boredom are the reasons people refer to using pornography [6, 7]. Furthermore, the disparity in pornography usage among women based on their birthplace may be attributed to the differences that exist in affective-sexual education and socialization around sexual roles between the Spanish and Latino American populations. The studies conducted with ethnic minorities show results in the same sense. Studies by Perry [29] analyze the association of race and sex with the use of pornography amongst the US population. The analyses show that African-American people were more likely to consume pornography than white people. In particular, black men were more likely to use pornography than all other race/gender combinations, with white women being those least likely to consume.

The limitations and strengths of this study need to be considered when interpreting our results. This is the first study in Spain to analyze the prevalence of using pornography and the factors associated with a representative sample of a young adult Spanish population. The cross-sectional design of the study does not enable to establish causal relationships, however, the analyzed variables are mostly socio-demographic, therefore, they are stable over time. Despite the analyzed sample being representative, it does not allow us to independently analyze the different categories of sexual attraction, which has led us to group homosexual and bisexual people into a single category of analysis. In relation to the birthplace, the percentage of Latin American immigrants included in our sample (11.9%), aligns closely with the estimated Latin American population of this particular age group residing in Spain (12.2%). The dependent variable we analyzed does not enable us to examine the frequency nor the type of pornography consumed nor whether or not it is violent pornography. These consumption characteristics are important given their association with affective-sexual health [20, 21, 46, 47].

## Conclusions

We can conclude that consuming pornography in Spain is a frequent practice among young adults that start watching it at a young age, especially men. Regarding women, use among homosexual and bisexual women and foreign women can be outlined. The results show the importance of introducing public health programmes on affective-sexual health focusing on a multicultural approach and considering people’s sex, sexual attraction and migratory status. There is a need to promote a critical attitude towards the use of pornography given the implication that it can have in constructing men and women’s sexuality, as well as in the inequalities between women and men.

## Data Availability

The datasets and material that have been produced during the current study are available from the corresponding author on reasonable request that guarantee their use according to the ethical procedures adopted in this project and participants’ informed consent documents content.
